# TACC3 promotes stemness and is a potential therapeutic target in hepatocellular carcinoma

**DOI:** 10.18632/oncotarget.4643

**Published:** 2015-06-25

**Authors:** Dong-Sheng Zhou, Hong-Bo Wang, Zhong-Guo Zhou, Yao-Jun Zhang, Qian Zhong, Li Xu, Yue-Hua Huang, Sai-Ching Yeung, Min-Shan Chen, Mu-Sheng Zeng

**Affiliations:** ^1^ Sun Yat-sen University Cancer Center, State Key Laboratory of Southern China, Collaborative Innovation Center for Cancer Medicine, Guangzhou, P. R. China; ^2^ Shandong Provincial Qianfoshan Hospital, Jinan, P. R. China; ^3^ Guangdong Provincial Key Laboratory of Liver Disease Research, The Third Affiliated Hospital of Sun Yat-sen University, Guangzhou, P. R. China; ^4^ Department of General Internal Medicine, Ambulatory Treatment and Emergency Care, University of Texas MD Anderson Cancer Center, Houston, TX, USA; ^5^ Department of Endocrine Neoplasia and Hormonal Disorders, University of Texas MD Anderson Cancer Center, Houston, TX, USA

**Keywords:** hepatocellular carcinoma, TACC3, stem cell

## Abstract

Transforming acidic coiled-coil protein 3 (TACC3) is essential for cell mitosis and transcriptional functions. In the present study, we first demonstrated that both TACC3 protein and mRNA levels were elevated in HCC tissue samples compared with non-cancerous tissue biopsies according to western blot analyses, immunohistochemistry (IHC) and quantitative real-time PCR (qRT-PCR) assays. Moreover, high TACC3 expression was positively correlated with poor overall survival (OS) and disease-free survival (DFS) (*p* < 0.001). Using HCC cell lines, we then demonstrated that either TACC3 knockdown or treatment with the potential TACC3 inhibitor KHS101 suppressed cell growth and sphere formation as well as the expression of stem cell transcription factors, including Bmi1, c-Myc and Nanog. Silencing TACC3 may suppress the Wnt/β-catenin and PI3K/AKT signaling pathways, which regulate cancer stem cell-like characteristics. Taken together, these data suggest that TACC3 is enriched in HCC and that TACC3 down-regulation inhibits the proliferation, clonogenicity, and cancer stem cell-like phenotype of HCC cells. KHS101, a TACC3 inhibitor, may serve as a novel therapeutic agent for HCC patients with tumors characterized by high TACC3 expression.

## INTRODUCTION

Hepatocellular carcinoma (HCC) is the ﬁfth most frequently diagnosed cancer and the third most common cause of cancer-related mortality in the world; therefore, HCC is a serious global health problem [1]. The incidence of HCC has been increasing in recent decades [2]. Despite improvements in HCC diagnosis and treatments, only 30% to 40% of HCC patients undergo curative therapy [3-5]. Intermediate- to advanced-stage HCC tumors are highly aggressive and either respond poorly to common therapies or do not respond at all. Therefore, new therapeutic strategies that are effective and well-tolerated are urgently required.

Targeted therapy, a newly emerging research area, has significantly changed the traditional treatment of cancer, including HCC, over the past 15 years [6-9]. Sorafenib, an oral multikinase inhibitor of the VEGF and PDGF receptors, has been confirmed in clinical trials to be effective and safe in patients with advanced HCC [2, 8, 10, 11]. Although all of these clinical trials were adjusted for advanced-stage tumors, the results were still not promising. Tumor cell stemness has been frequently investigated as an emerging new area of focus. A large number of studies have indicated that cancer stem cells (CSCs), which are present in the original tumor, have the capacity to self-renew and give rise to a variety of differentiated cells [12]. CSCs play key roles in tumorigenesis, recurrence and metastasis. Therefore, inhibiting CSC functions will reduce tumor proliferation and likely cure malignant tumors. Signaling pathways such as the Wnt, Notch, Shh and PI3K/AKT/mTOR pathways play important roles in the regulation of HCC stem cells [13-16]. Thus, identifying factors that block one or more of these signaling pathways might lead to the inhibition of cancer stem-like capabilities.

Transforming acidic coiled-coil protein (TACC) family members are characterized by a highly conserved C-terminal coiled-coil domain and comprise centrosome- and microtubule-associated proteins. To date, TACCs have been considered adapters that interact with the spindle and centrosome apparatus to promote centrosome integrity, microtubule assembly and spindle stability during mitosis [17-21]. In addition to their function in mitosis, TACCs are involved in controlling cell growth, differentiation and transcriptional regulation [19, 20, 22-24]. As a member of the TACC family, TACC3, located on chromosome 4p16.3, also stabilizes and organizes the mitotic spindle to allow for proper chromosomal segregation [25, 26]. Phosphorylation of the TACC3-chTOG complex on Ser-558 by Aurora and integrin-linked kinase (ILK) is the key mechanism involved in maintaining spindle microtubule growth and stability [18, 27-33]. Clathrin heavy chain (CHC) serves as an adaptor protein to recruit phosphorylated TACC3 to the spindle during mitosis [34]. Angelica et al recently discovered an FGFR-TACC fusion protein in a small subset of glioblastoma multiforme (GBM) tumors. The fusion protein, which localizes to mitotic spindle poles, has constitutive kinase activity, induces mitotic and chromosomal segregation defects and triggers aneuploidy [35]. The fusion gene, which has been defined as an oncogene, has also been identified in nasopharyngeal carcinoma, lung adenocarcinoma and bladder cancer [36-38]. TACC3 may also interact with the signal transducer and activator of transcription 5 (STAT5), aryl hydrocarbon receptor nuclear translocator (ARNT) and Friend of GATA-1 (FOG-1) [24, 39, 40], suggesting a potential role in the regulation of gene transcription. TACC3 inhibits Notch signaling by interacting with Notch4/Int3 [22]. TACC3 also promotes ERK and PI3K/AKT signaling, which induces the epithelial-mesenchymal transition (EMT), leading to cervical cancer initiation [41]. Considerable evidence exists supporting the involvement of TACC3 dysregulation in tumorigenesis. However, TACC3 expression varies in different cancers. For example, TACC3 expression is increased in GBM, non-small cell lung cancer (NSCLC), and breast cancer but decreased in ovarian and thyroid cancers [31, 42-45]. To date, the role of TACC3 in HCC has remained elusive. In the present study, we first assessed TACC3 expression in HCC and then elucidated the molecular mechanisms underlying the TACC3-mediated regulation of CSC-like characteristics. Taken together, these data suggest that TACC3 might be a new therapeutic target for HCC.

## RESULTS

### TACC3 expression was increased in HCC cells and tumor tissues

**Figure 1:**
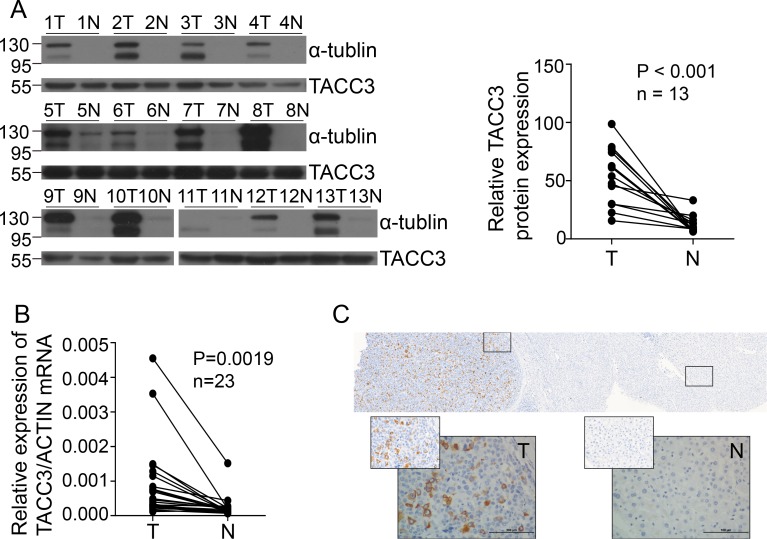
TACC3 expression is increased in HCC patients **A.** TACC3 and α-tubulin expression in tumor tissues (T) compared with adjacent matched normal tissues (N), as assessed by western blotting. (*n* = 13 paired, *p* < 0.001, paired Wilcoxon signed-rank test). **B.** TACC3 mRNA levels were analyzed using qRT-PCR assays and normalized to the housekeeping gene β-actin (*n* = 23 paired, *p* = 0.0019). N, matched normal tissue; T, tumor tissue. **C.** TACC3 expression in 237 HCC patients’ tissues analyzed by IHC. The left image shows TACC3 expression in the tumor tissue and adjacent non-tumor tissue. The right two images show the different intensities of expression. All images were magnified 200× and 400×.

To investigate TACC3 expression in HCC, TACC3 mRNA and protein levels were analyzed by qRT-PCR assays and western blot analysis in the immortalized liver cell line LO2 and in a panel of HCC cell lines (SK-Hep-1, SMMC-7721, Bel-7402, MHCC-97L, QGY-7703, and Huh7). TACC3 expression was clearly higher in the HCC cell lines than in the immortalized liver cell line LO2 (Figure 3A, 3B). We further examined TACC3 expression in HCC patients. TACC3 protein expression (92.2%, 12/13) was increased in the HCC tumor tissues compared with the non-cancerous samples (Figure 1A). *TACC3* mRNA levels (95.2%, 21/23) were also elevated in the tumor tissues compared with the counterpart non-cancerous tissues (Figure 1B). To further examine TACC3 expression in fresh HCC tissues *in situ*, IHC staining was employed. As shown in Figure 1C, TACC3 protein was expressed predominantly in the plasma membrane of HCC cells in the tumor regions (T), whereas TACC3 was only occasionally expressed in the liver cells of the adjacent non-cancerous tissue (N). Taken together, these data demonstrated that TACC3 expression was increased in HCC tumor tissues.

### High TACC3 levels were correlated with a poor prognosis in HCC patients

**Figure 2:**
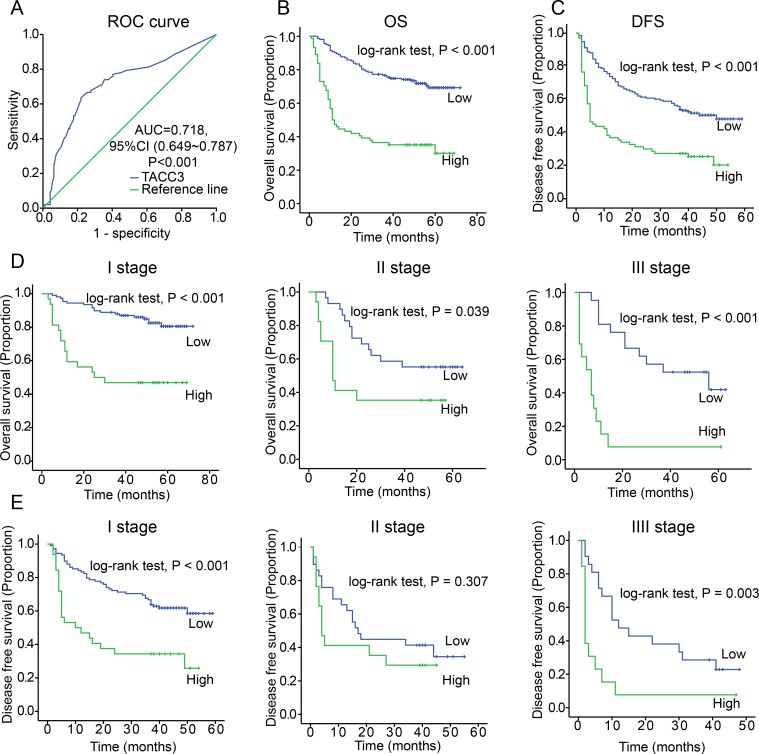
Increased TACC3 expression is correlated with a poor prognosis of HCC **A.** The Youden's index threshold was calculated by ROC curve analysis. (AUC = 0.718, threshold = 0.4). **B.** TACC3 expression was associated with OS according to Kaplan-Meier analysis. (*n* = 237, *p* < 0.001). **C.** TACC3 expression was associated with DFS according to Kaplan-Meier analysis. (*n* = 237, *p* < 0.001). **D.** Subgroup analysis for OS based on TNM staging (Stage I, *p* < 0.001; Stage II, *p* = 0.039; Stage III, *p* < 0.01) **E.** Subgroup analysis for DFS based on TNM staging (Stage I, *p* < 0.001; Stage II, *p* = 0.307; Stage III, *p* = 0.003).

To identify the relationship between TACC3 expression and clinicopathological variables, 237 HCC specimens were subjected to IHC staining (Figure 1C). High TACC3 expression was correlated with poorly differentiated HCC tissues (Table 1). The median follow-up period of the entire studied cohort was 49 months, and the median OS was 40 months. The 1-, 3-, and 5-year OS rates were 76.8%, 64.1%, and 59.5%, respectively. According to the ROC curve analysis, the TACC3 expression cut-off value was 0.4 (Figure 2A); a score exceeding 0.4 was defined as high expression. Of the 237 HCC tumors, 74 (31.2%) had high TACC3 expression. Tumor size showed the most significant correlation with TACC3 expression (Table 1, *p* < 0.001).

**Table 1:** Correlation between TACC3 and clinicopathological features

Clinicopathological features	TACC3 expression level (n)			
Number	Up-regulated	Down-regulated	*p* value
Age (years)				
Younger than 50	126	40	86	0.776
Older than 50	111	34	77	
Gender				
Male	211	64	147	0.401
Female	26	10	16	
AFP				
Lower than 400 ng/ml	123	27	97	0.001
Higher than 400 ng/ml	114	47	66	
Microvascular invasion				
Absent	197	50	147	<0.001
Present	40	24	16	
Tumor size				
Less than 5 cm	102	16	86	<0.001
More than 5 cm	135	58	77	
Tumor nodules				
Single	176	52	124	0.346
Multiple	61	22	39	
pTNM stage				
Early stage (I-II)	140	32	108	0.001
Advanced stage (III-IV)	97	42	55	
Differentiation				
I-II	178	143	35	<0.001
III-IV	59	20	39	
Five-year recurrence				
Yes	137	56	81	< 0.001
No	100	18	82	

Abbreviations: AFP=alpha-fetoprotein

According to the Kaplan-Meier analysis, patients with higher TACC3 expression suffered from decreased OS and DFS (*p* < 0.001, Figure 2B, 2C). Stratification analysis based on the TNM staging revealed that higher TACC3 levels suggested a poorer prognosis in early-intermediate stages (Figure 2D, 2E). No correlation was found between TACC3 expression and stage IV HCC (Supplementary Figure 2). According to the multivariate analysis, TACC3 expression (HR = 2.267, *P* < 0.001), tumor size (HR = 2.358, *p* = 0.003), microvascular invasion (HR = 2.527, *p* < 0.001), and TNM staging (HR = 1.272, *P* = 0.035) were independent predictors of survival.

### TACC3 knockdown suppresses the proliferation and clonogenicity of HCC cells

**Figure 3:**
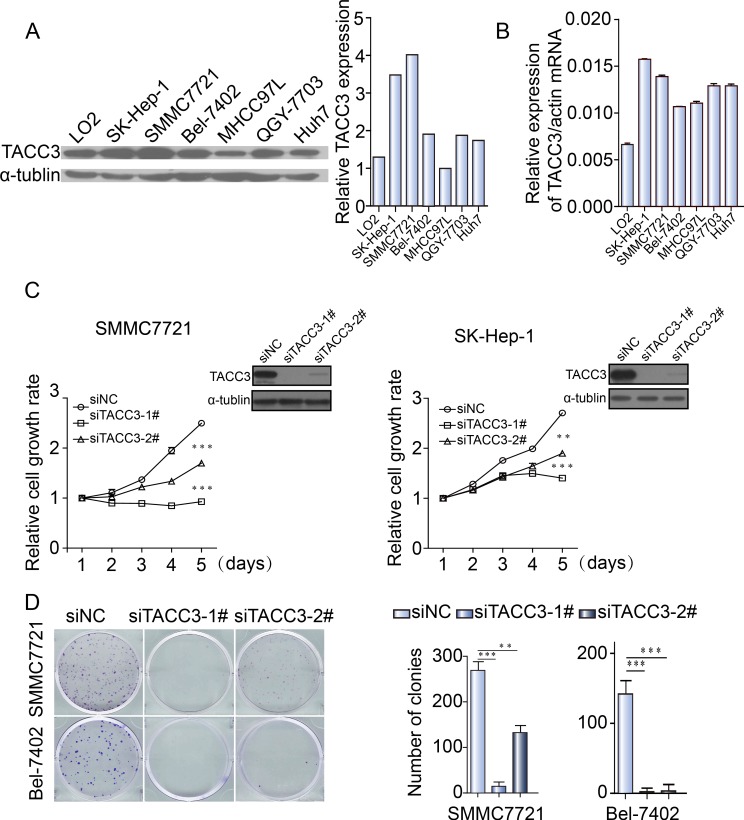
TACC3 knockdown suppresses the proliferation and clonogenicity of HCC cells **A.** TACC3 protein expression in different HCC cell lines, as assessed by western blotting. **B.** TACC3 mRNA levels in different HCC cell lines, as assessed by qRT-PCR. **C.** The effect of TACC3 knockdown with siRNA and NC was verified by western blotting 48 hours after transfection (right top corner), and cell viability was assessed with an MTT assay at the indicated times. **D.** Colony formation assays of SMMC-7721 and SK-Hep-1 cells infected with NC or TACC3-targeted siRNAs (*p < 0.05, **p < 0.01, ***p < 0.001).

To explore the potential role of TACC3 in HCC tumorigenesis, TACC3 expression was knocked down in SMMC-7721, SK-Hep-1, Bel-7402 and Huh-7 cells with two distinct siRNA duplexes. Western blotting was used to validate the knockdown efficiency. As depicted in Figure 3C, TACC3 expression was nearly abolished in the siTACC3 transfectants. Viability was assessed at the indicated times with an MTT assay. Compared with the control, the siTACC3 transfectants displayed significantly decreased proliferation (Figure 3C and Supplementary Figure 1). A clonogenicity assay was then performed to evaluate the effect of TACC3 knockdown on the clonogenicity of the HCC cells. Both the size and number of siTACC3 transfectants were dramatically decreased compared with the SiNC cells (Figure 3D and Supplementary Figure 1).

### TACC3 knockdown suppressed sphere formation and stem cell markers *in vitro*

**Figure 4:**
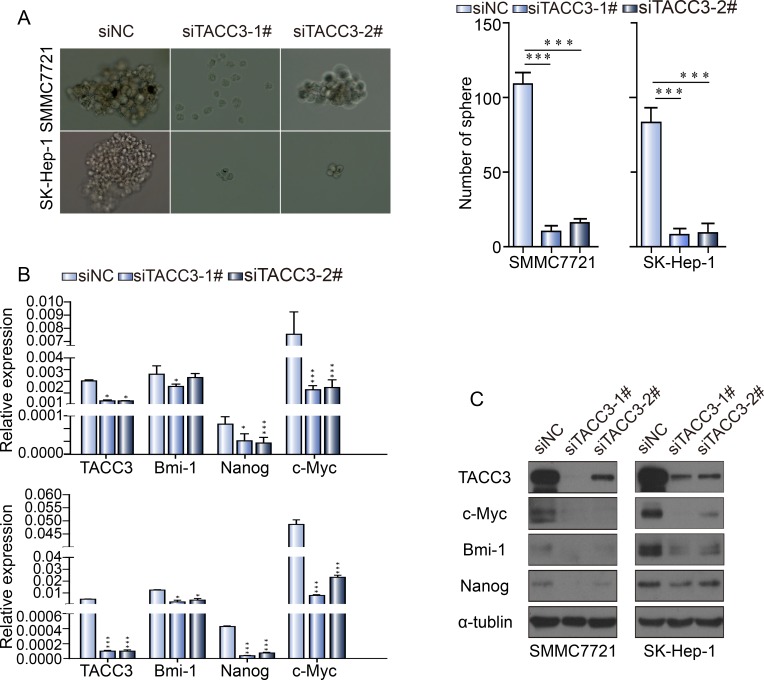
TACC3 knockdown suppresses sphere formation and stem cell marker expression by HCC cells **A.** TACC3 knockdown suppressed sphere formation by SMMC-7721 and SK-Hep-1 cells. SMMC-7721 and SK-Hep-1 cells transfected with siRNAs or NC were grown in tumor sphere-forming medium for 7 days. Colonies were photographed at 200× magnification and were counted at 40× magnification. **B.** The effect of TACC3 knockdown on SCTF expression normalized to β-actin. **C.** The effect of TACC3 knockdown on stem cell-related protein expression, as assessed by western blotting (*p < 0.05, **p < 0.01, ***p < 0.001).

**Figure 5:**
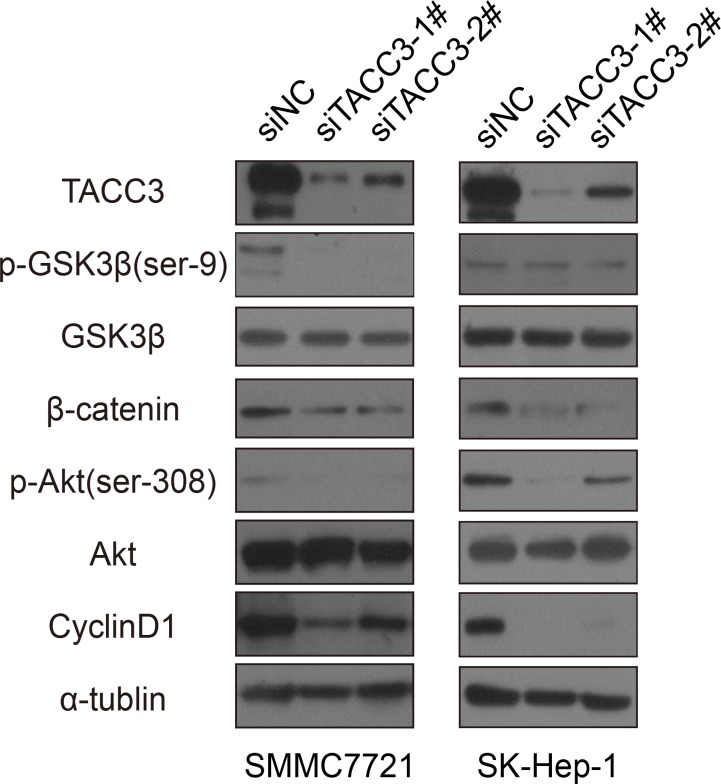
Potential mechanism for the stem cell-suppressing activity of TACC3 The activity of downstream signaling molecules was analyzed by western blotting; α-tubulin was used as a loading control.

To investigate the effect of TACC3 on stem cell-like capabilities, a sphere formation assay was performed. After transfection with the siRNA duplexes, SMCC-7721 and SK-Hep-1 cells were cultured in specific sphere-forming medium containing EGF and β-FGF for 2 weeks. The numbers and sizes of spheres in the siTACC3-knockdown cells were significantly decreased compared with the NC-transfected cells (Figure 4A). Western blot and qRT-PCR assays were used to assess whether TACC3 down-regulation was associated with changes in stem cell transcription factors (SCTFs). Bmi1, c-Myc and Nanog were decreased concurrently with TACC3 down-regulation (Figure 4B, 4C). SOX2 was undetectable by both qRT-PCR and western blotting. TACC3 is involved in the Wnt/β-catenin and AKT pathways [41], which are involved in the EMT in cervical cancer. TACC3 knockdown suppressed tumor stem cell-like characteristics through the Wnt/β-catenin and PI3K/AKT signaling pathways. We therefore examined whether TACC3 participated in Wnt/β-catenin pathway activation in the HCC cell lines. Compared with the negative control, TACC3 down-regulation led to decreased GSK3β (Ser-9) phosphorylation and β-catenin down-regulation, whereas total GSK3β-levels remained unchanged (Figures 4C and 5). β-catenin/TCF activation leads to the increased expression of downstream target genes such as c-Myc and cyclin D1 [47-50]. Accordingly, TACC3 knockdown led to decreased c-Myc and cyclin D1 expression (Figures 4C and 5). These data suggested that the knockdown of TACC3 suppressed the Wnt/β-catenin signaling pathway, possibly contributing to the inhibition of stem cell processes. Accumulating evidence indicates that GSK3β is a downstream target of the PI3K/AKT signaling pathway [51]. Therefore, we examined whether TACC3 down-regulation inhibited AKT activity. As shown in Figure 5, TACC3 down-regulation reduced AKT phosphorylation at Ser-308, whereas total AKT levels remained stable. These results indicated that TACC3 down-regulation reduced AKT activation. Together, these data suggested that TACC3 down-regulation reduced stem cell-like traits at least in part through inhibition of the PI3K/AKT and Wnt/β-catenin signaling pathways (Figure 5).

**Table 2:** Analyses of prognostic factors for overall survival

Variables	Univariate analysis	Multivariate analysis	
	*p*-value	Hazard ratio(95% CI)	*p*-value
Age (<50 y/≥50 y)	0.452		
Gender (M/F)	0.494		
PLT Count (≤100×10^9^/L/>100×10^9^/L)	0.222		
ALT (≤40 U/L/>40 U/L)	0.914		
AST (≤45 U/L/>45 U/L)	<0.001		
ALP (≤110 U/L/>110 U/L)	0.013		
AFP (≤400 ng/mL/>400 ng/mL)	<0.001		
AFU (≤40 U/L/>40 U/L)	0.001		
Maximum diameter of largest lesion(<5 cm/≥5 cm)	<0.001	2.358 (1.334-4.166)	0.003
Number of lesions (single/multiple)	0.125		
Microvascular invasion (absent/present)	<0.001	2.527 (1.504-4.254)	<0.001
Child-Pugh grade (A/B)	0.009		
TNM (I/II/III/IV)	<0.001	1.272 (1.018-1.590)	0.035
Differentiation	0.002		
TACC3 (≤0.4/>0.4)	<0.001	2.267 (1.455-3.533)	<0.001

Abbreviations: PLT=platelet, ALT=alanine aminotransferase, AST=aspartate aminotransferase, ALP=alkaline phosphatase, AFP=alpha-fetoprotein, AFU=alpha-L-fucosidase.

### The TACC3 protein inhibitor KHS101 suppressed cell proliferation and the stem-like phenotype *in vitro*

**Figure 6:**
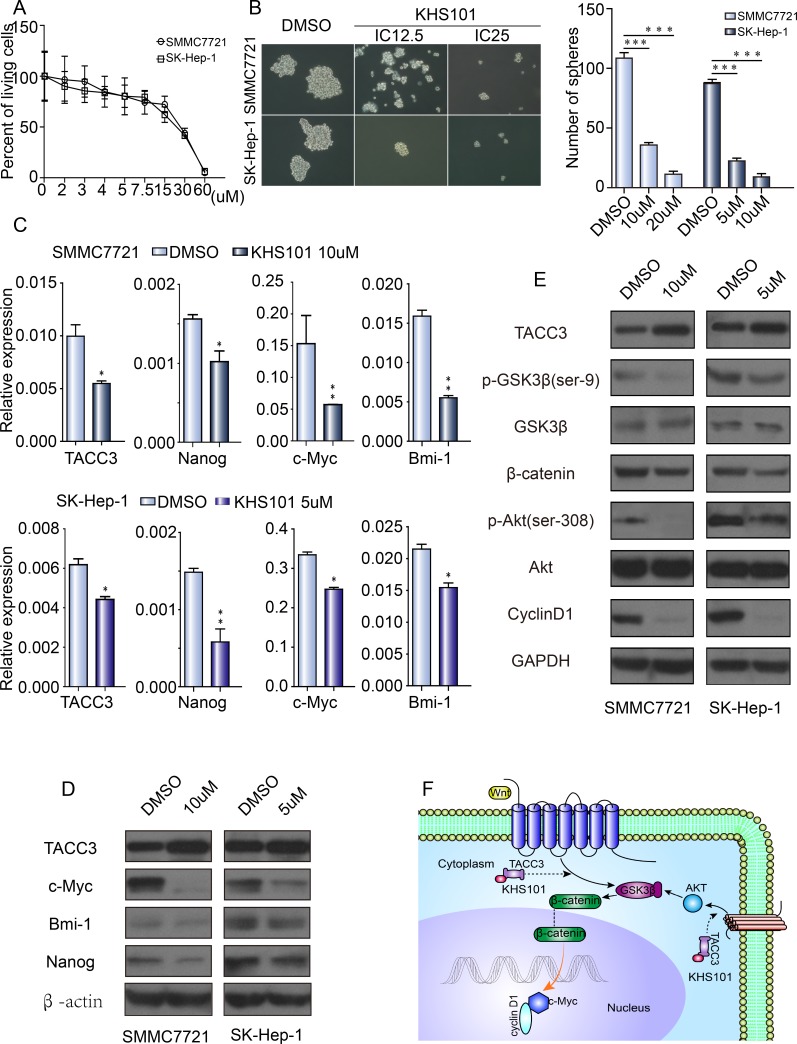
KHS101, a TACC3 inhibitor, suppresses stemness **A.** The IC50 of KHS101 in SMMC-7721 (40 μM) and SK-Hep-1 (20 μM) cells. **B.** KHS101 suppressed sphere formation by HCC cells. SMMC-7721 and SK-Hep-1 cells treated with DMSO or KHS101 were grown in tumor sphere-forming medium for 7 days. Colonies were photographed at 200× magnification and counted at 40× magnification. **C.** The effect of KHS101 on SCTF expression, normalized to β-actin. **D.** The effect of KHS101 on stem cell-related protein expression, as determined by western blotting. **E.** The effect of KHS101 on the expression of Wnt/β-catenin and PI3K/AKT signaling pathway proteins, assayed by western blotting and normalized to GAPDH (*p < 0.05, **p < 0.01, ***p < 0.001). **F.** Diagram depicting upstream and downstream signals of TACC3 that might contribute to Wnt/β-catenin and PI3K/AKT signaling pathway activation.

KHS101, a small molecule known for its role in neuronal differentiation, binds to the TACC3 protein. Likely through a functional link between KHS101 and the TACC3-ARNT2 axis, KHS101-mediated interference with TACC3 accelerates neurogenesis and concomitantly suppresses proliferation. However, whether KHS101 suppresses liver CSC-like characteristics is unknown. SMMC-7721 and SK-Hep-1 cells were cultured with different KHS101 concentrations (40 μM and 20 μM, respectively, Figure 6A) to determine the IC50 values. The IC12.5 and IC25 concentrations were then used to test the interference in HCC cell lines. After KHS101 treatment, SMCC-7721 and SK-Hep-1 cells were cultured in specific F12 sphere-forming medium for 1 week. The number and size of spheres were significantly decreased after treatment with KHS101 compared with the control (DMSO) treatment (Figure 6B). As illustrated in Figure 6B, sphere formation was dependent on the KHS101 concentration. qRT-PCR and western blot assays were used to assess whether KHS101 was associated with changes in SCTFs. Bmi1, c-Myc, and Nanog were all decreased in the presence of KHS101 (Figure 6D). Western blot analysis was performed to investigate the mechanism by which KHS101 inhibits sphere formation. Compared with the control (DMSO), KHS101 decreases the expression of p-AKT, p-GSK3β and β-catenin, as well as the expression of the downstream markers c-Myc and cyclin D1 (Figure 6E). Thus, TACC3 may mediate the development of liver CSC-like characteristics through the PI3K/AKT and Wnt/β-catenin signaling pathways.

## DISCUSSION

The clinical complexity, genetic diversity and molecular heterogeneity of HCC make this cancer a worldwide health problem and hamper the progress of therapeutic development. This study explored increased TACC3 expression in HCC and the dynamic interplay between stem cell-like characteristics and signaling pathways to assess whether TACC3 is a novel target for HCC therapy.

As discussed above, TACC3 levels in various cancers are controversial and are correlated with the type of cell, organ or carcinoma [52], and the molecular mechanisms of TACC3 remain elusive. In the present study, we determined that TACC3 might be an oncogene that plays an important role in tumorigenesis, differentiation, amplification and metastasis in HCC. Based on our data, TACC3 expression was increased, as measured by both western blotting and qRT-PCR assays. As assessed by IHC staining, TACC3 expression in HCC specimens was clearly higher than that in non-cancerous tissue (Figure 1C). Furthermore, according to univariate and multivariate analyses, TACC3 was an independent predictor of prognosis because higher TACC3 expression corresponded with a poorer OS and DFS. The subgroup analysis based on TNM staging revealed that TACC3 only accurately predicted the prognosis in early- to intermediate-stage tumors. There are three likely reasons for this phenomenon: (1) stage IV patients accounted for only a small proportion of the total patient population (17/237), which might skew the results; (2) stage IV tumors usually occur with hepatic dysfunction, which may affect the prognosis; and (3) TACC3 levels were absolutely correlated to the stage of carcinoma. Additionally, high TACC3 expression was positively correlated with poor differentiation, microvascular invasion, and, in particular, tumor size. Taken together, our data revealed that TACC3 might serve as an oncogene in HCC. High TACC3 levels predicted a poor prognosis, highlighting the potential use of a TACC3 inhibitor as an HCC therapy.

An interesting phenomenon was observed in the IHC analysis. The distribution of TACC3 expression was sporadic; this protein accumulated at the border of neoplastic cell nests and was sometimes present in normal tissues. TACC3 expression might correlate with the EMT, as suggested by Geun [41]. In addition to the EMT, TACC3 may also affect tumor stem cell-like capabilities. Based on this hypothesis, a sphere formation assay and related experiments were performed. TACC3 down-regulation prominently suppressed CSC phenotypes and critical SCTFs such as Bmi1, c-Myc, and Nanog. The polycomb-group (PcG) gene Bmi1 plays a critical role in the self-renewal of a range of somatic stem cells, including hepatic stem cells [53, 54]. The transcription factor Nanog is essential for ESC self-renewal, protein expression, differentiation and tumorigenesis [55, 56]. Nanog also predicts prognosis and regulates human tumor development [57, 58]. c-Myc is a key factor for stem cell self-renewal and differentiation during tumor development and oncogenic processes; additionally, c-myc regulates cell cycle progression and apoptosis [59, 60]. Notably, the PTEN/PI3K pathway can directly repress c-Myc. Specifically, p53 represses c-Myc transcription by directly binding the c-Myc promoter [61], whereas the downstream PI3K pathway components inhibit c-Myc translation and promote protein degradation [62, 63]. In the present study, both western blot and qRT-PCR assays demonstrated that TACC3 knockdown suppressed stem cell-like markers, indicating that TACC3 may be involved in certain signaling pathways that promote stem cell-like traits.

c-Myc and cyclin D1 are two key downstream targets of the Wnt/β-catenin pathway. The expression of these two markers decreased in conjunction with decreased TACC3 expression, indicating that the Wnt/β-catenin signaling pathway might be the molecular mechanism by which TACC3 promotes stem cell traits. Wnt/β-catenin signaling is a crucial stem cell regulator and plays various important roles in many cancers. Aberrant Wnt/β-catenin signaling leads to a wide range of human pathologies. When the receptors of the Frizzled and LRP families (Lrp5/6) are bound to ligands on the cell surface, the signal is transduced to β-catenin, which is the central player located in cytoplasm. Mutant forms of β-catenin are associated with many cancers, including HCC. β-catenin stability is regulated by the destruction complex, which includes the tumor suppressors adenomatous polyposis coli (APC) and axin. If the receptor is not bound to Wnt, then CKI and GSK3, two kinases residing in the complex, phosphorylate a set of conserved Ser and Thr residues at the amino terminus of β-catenin, thereby recruiting a b-TrCP-containing E3 ubiquitin ligase, which targets β-catenin for proteasomal degradation. If the receptor binds Wnt, GSK3 is phosphorylated, leading to β-catenin accumulation. Activated β-catenin travels into the nucleus, where it activates the N termini of Tcf/Lef family DNA-binding proteins. The c-myc gene has been identified as a direct target of LEF-1/TCF proteins, which are repressed by wild-type APC and activated by β-catenin in colon cancer cells [48]. Cyclin D1, which is regulated by β-catenin, contributes to tumor progression. In this study, knocking down TACC3 increased p-GSK3β levels, which then suppressed β-catenin activation, leading to reduced c-Myc and cyclin D1 levels. Finally, stem cell-like capabilities were decreased. β-catenin activation by the PI3K/AKT pathway has also been reported in HepG2 and HeLa cells. We obtained consistent results by verifying the expression of AKT. Taken together, these results indicate that TACC3 can affect stem cell-like traits, partially by affecting the Wnt/β-catenin and PI3K/AKT signaling pathways.

The small molecule KHS101 was recently found to bind to the TACC3 protein. By interacting with the TACC3-ARNT2 axis, KHS101 inhibited cell cycle progression in a similar manner as TACC3 siRNA. However, the molecular mechanisms downstream of TACC3 have not been determined. In this study, KHS101 significantly suppressed the proliferation and stem cell-like traits, as well as the expression of stem cell markers, of both SMMC-7721 and SK-Hep-1 cells via the Wnt/β-catenin and PI3K/AKT pathways. Notably, TACC3 expression was slightly increased. Mechanistically, KHS101 likely binds to TACC3 in the cytoplasm to increase TACC3 protein synthesis. Taken together, these results indicate that TACC3 might be a molecular target for clinical HCC therapy.

In summary, we investigated the expression and potential role, as well as the underlying molecular mechanisms, of TACC3 in HCC. Our data revealed that TACC3 up-regulation was correlated with a poor prognosis and highlighted the potential use of a TACC3 inhibitor in clinical HCC therapy.

## MATERIALS AND METHODS

### Patients and tissue specimens

Paraffin-embedded HCC specimens (*n* = 237) and fresh frozen HCC tissues (*n* = 23) from cases that were histologically confirmed and did not undergo neo-adjuvant treatment were obtained from the Cancer Center of Sun Yat-sen University in Guangzhou. Written informed consent was obtained from each patient, and the study was approved by the Institute Research Ethics Committee at the Cancer Center.

### Cell culture

The HCC cell lines (LO2, SK-Hep-1, SMMC-7721, Bel-7402, MHCC-97L, QGY-7703, and Huh7) were cultured in Dulbecco's modified Eagle's medium (DMEM; Gibco, Carlsbad, CA, USA) supplemented with 10% fetal bovine serum (FBS; Gibco) in a humidified 5% CO_2_ incubator at 37°C.

### Quantitative RT-PCR analysis

RNA was extracted from fresh tissue specimens and HCC cell lines using TRIzol reagent (Invitrogen, Grand Island, NY, USA). Complementary DNA (cDNA) was synthesized using a reverse transcriptase kit (Invitrogen). Primers for the quantitative RT-PCR (qRT-PCR) assays are presented in Supplementary Table 1. Three independent experiments were performed to verify the quantitation of the data.

### Western blot analysis

Western blotting was performed as previously described [46]. The primary and conjugated secondary antibodies used are listed in Supplementary Table 2.

### Immunohistochemical analysis

The paraffin-embedded HCC samples were cut into 4-μm-thick sections. Immunohistochemical staining was performed as previously described [46]. Briefly, after deparaffinizing and rehydrating the sections and blocking endogenous peroxidase activity, the sections were boiled in a citrate antigen retrieval solution (pH = 6.0) for 2 min in an electric pressure cooker for antigen retrieval. The sections were then incubated overnight with a rabbit polyclonal anti-TACC3 antibody (diluted 1:800, Abcam, USA) at 4°C, followed by incubation with a secondary antibody for 30 min at 37°C. Finally, the sections were dehydrated and mounted.

Each section was evaluated with an automated image analyzer by a pathologist who was blinded to the clinical status of the patients. TACC3 expression was evaluated according to the intensity and extent of staining. The staining intensity was scored using the following system: 0, negative staining; 1, weak staining; 2, moderate staining; and 3, strong staining. The extent of staining was scored as the percentage of positive cells. A final immunoreactivity score (IRS) was given for each sample, defined as the intensity score multiplied by the extent score.

### siRNA transfection

siRNA oligo-ribonucleotides were purchased from RiboBio (RiboBio, Guangzhou, China); detailed information is presented in Supplementary Table 3. The negative control (NC) RNA duplex for the siRNA was not homologous to any human genome sequences. SMMC-7721 and SK-Hep-1 cells (2×10^5^) were seeded into 6-well plates, incubated for 24 h and then transfected with 12.5 nM of the RNA duplex and 5 μL of Lipofectamine RNAiMAX (Invitrogen, Grand Island, NY, USA) according to the manufacturer's instructions. The cells were harvested for further experiments after 48 or 72 h.

### MTT assay

An MTT [3-(4,5-dimethyl-2-thiazolyl)-2,5-diphenyl-2-H-tetrazolium] assay was used to measure cell viability. After transfection with siNC, siTACC3-1#, or siTACC3-2# for 48 h, SMMC-7721 and SK-Hep-1 cells (3,000 cells/well) were plated in sextuplicate in a 96-well plate and cultured with DMEM containing 10% FBS. At the indicated time points, 20 μL of 5 mg/mL MTT was added to each well, and the cells were incubated for 4 h at 37°C. Then, 150 μL of 100% dimethylsulfoxide (DMSO) was used to dissolve the precipitates. The absorbance was measured at 490 nm with a SpectraMax M5 (Molecular Devices, Sunnyvale, USA) plate reader.

### Colony formation assay

Forty-eight hours after transfection with siNC, siTACC3-1#, or siTACC3-2#, SMCC-7721, Bel-7402 (each at 500 cells/well) and SK-Hep-1 (2,000 cells/well) cells were plated in triplicate in 6-well plates and cultured for 10 days. After fixation with methanol for 15 min, the colonies were stained with 0.5% crystal violet in 20% methanol for 15 min and counted. Data were acquired from three independent experiments.

### Sphere formation assay

After transfection with either siNC, siTACC3-1#, or siTACC3-2# for 48 h and treatment with various concentrations of KHS101 (10 μM, 20 μM) for 48 h, SMCC-7721 and SK-Hep-1 cells (500 cells/well) were plated in triplicate in 6-well plates with tumor sphere medium containing serum-free DMEM/F-12, 10 ng/mL human recombinant bFGF and 10 ng/mL EGF for 7 days. Colonies were photographed at 200× magnification and were counted at 40× magnification

### Statistical analysis

A two-tailed Mann-Whitney U test was used to analyze the differences between groups. Receiver operating characteristic (ROC) curve analysis was used to identify the cut-off value for TACC3. A chi-squared test was used to analyze the relationship between TACC3 expression and clinicopathological characteristics. The Kaplan-Meier method was used to calculate the OS and DFS and to conduct sub-analyses according to the TNM staging, and comparisons were performed using the log-rank test. The prognostic variables used in predicting the OS were assessed by multivariate Cox proportional hazards regression analysis. Variables that were significant in the univariate analysis were subsequently tested with the multivariate Cox proportional hazard model. All of the statistical tests were two-sided, and differences with *p* < 0.05 was considered statistically significant. All of the statistical tests were performed using SPSS 19.0 statistical software (SPSS Company, Chicago, Illinois, USA).

## SUPPLEMENTARY MATERIAL TABLES AND FIGURES


