# Supplementary material for: TACC3 promotes stemness and is a potential therapeutic target in hepatocellular carcinoma
Source: Oncotarget. 2015 Jun 25;6(27):24163–77. doi: 10.18632/oncotarget.4643 (PMC4695177; doi:10.18632/oncotarget.4643)
Supplement: Supplementary file 1 [file oncotarget-06-24163-s001.pdf]

TACC3 promotes stemness and is a potential therapeutic target in hepatocellular carcinoma

Supplementary Material

Supplementary table 1 sequence of primers

Name	Sense primer(5'- 3')	Antisense primer(5'- 3')
TACC3	CCTCTTCAAGCGTTTTGAGAAAC	GCCCTCCTGGGTGATCCTT
BMI1	TGGCTCGCATTCATTTTCTG	TGTGGCATCAATGAAGTACCCT
MYC	GGAGGCTATTCTGCCCATTTG	CGAGGTCATAGTTCCTGTTGGTG
NANOG	CTAAGAGGTGGCAGAAAAACA	CTGGTGGTAGGAAGAGTAAAGG
KLF4	CACAAAGAGTTCCCATCTCAAGGC	CGGTAGTGCCTGGTCAGTTCATC
SOX2	CGAGTGGAAACTTTTGTCGGA	TGTGCAGCGCTCGCAG
OCT4	GTGGAGAGCAACTCCGATG	TGCTCCAGCTTCTCCTTCTC
β -actin	CGCGAGAAGATGACCCAGAT	GGGCATACCCCTCGTAGATG

Supplementary table 2 Reagents information

Name	Company	serial number
TACC3	abcam	ab134154
p-AKT(Thr308)	cell signaling	SC-16646-R
AKT	cell signaling	#9272S
p-GSK3β(ser9)	cell signaling	9336S
GSK3β	cell signaling	#9315S
β-catenin	Santa Cruz	sc-53483
c-MYC	cell signaling	#5605S
cylin D1	cell signaling	#2978S
Nanog	cell signaling	#4903S
Bmi-1	self-manufactured	S2608-2
SOX2	cell signaling	#3579S
α-tublin	sigma	T6199
GAPDH	Santa Cruz	sc-32233
β-actin	abcam	ab8226

Supplementary table 3 Human TACC3 siRNA sequence from Ribobiocompany

Name	siTACC3-#1	siTACC3-#2
Serial number	siG14313141600	siG141024100430
Target sequence	CCACAGATCTGAACTCCAT	GGATTACCTGGAGCAGTTT
Sense(5'-3')	CCACAGAUCUGAACUCCAUdTdT	GGAUUACCUGGAGCAGUUUdTdT
Antisense(5'-3')	AUGGAGUUCAGAUCUGUGGTdTd	AAACUGCUCCAGGUAAUCCTdTd

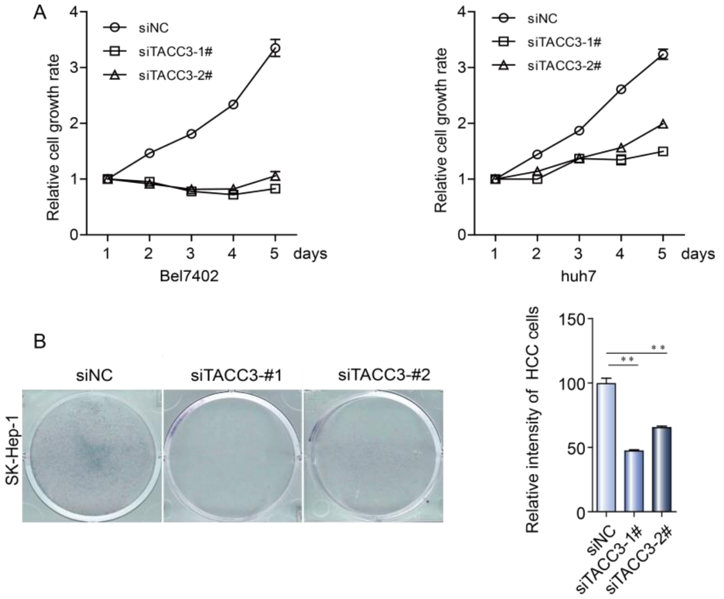

Supplementary Figure 1: Knockdown of TACC3 suppresses the proliferation and clonogenicity of HCC

The SK cell line didn’t show very well in colony formation assay, so this present study investigatedother cell lines to varied the effect of TACC3. (A)Cell viability was determined by MTT assay(B)Forty-eight hours after transfection with siNC, siTACC3-1#, siTACC3-2#, 20000 SK Cells were plated in triplicate in 6-well plates and cultured for 10 days. Figures showed that cells were decreased compared to NC. (*$p< 0.05$, **$p< 0.01$, ***$p< 0.001$)

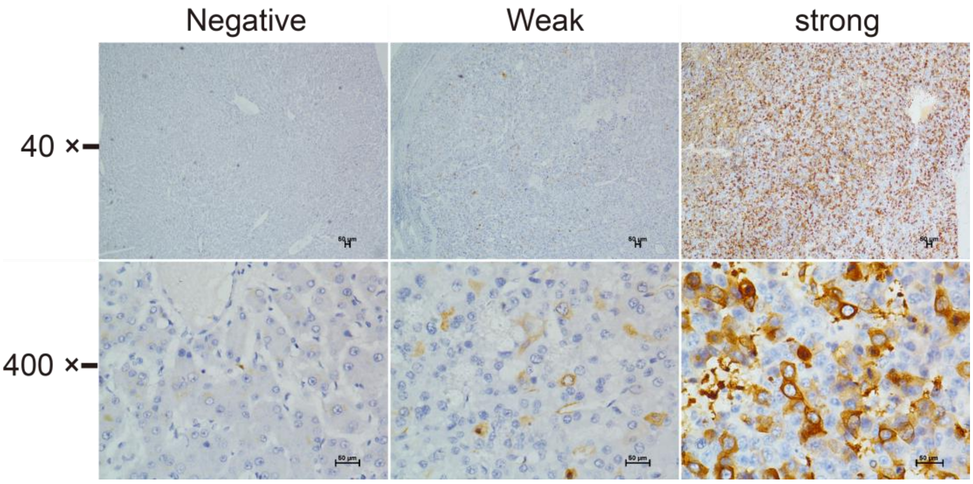

Supplementary Figure 2: Expression of TACC3 in HCC tissues by IHC

The representativeof the figure showed the different staining intensity in HCC tissues. negative staining of TACC3 in cytoplasm 4×, 400×; weak staining of TACC3 in cytoplasm 4×, 400×; strong staining of TACC3 in cytoplasm 4×, (J) 400×.We defined negative and weak staining as the “low expression” and strong staining as “high expression” using ROC curve.

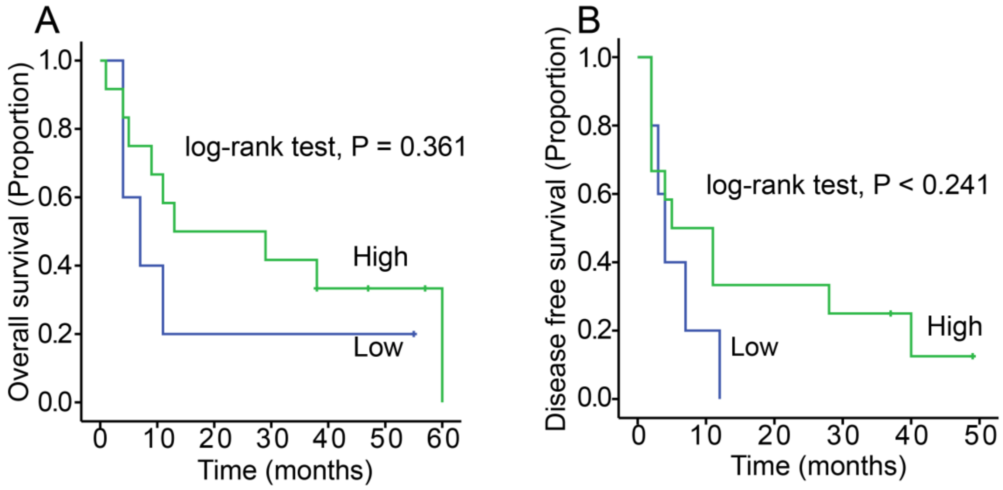

Supplementary Figure 3: Subgroup analysis based on stage IV for OS and DFS

The relationship between the expression of TACC3 and OS(A) or DFS(B) in stage IV had no significance. (OS, p=0.361; DFS, p<0.241, respectively) The probably elaborationwas as follows: 1. Patients recruited in stag IV occupied a little proportion, which made choice bias. 2. Tumors in Stag IV accompanied dysfunction of hepatocyte.

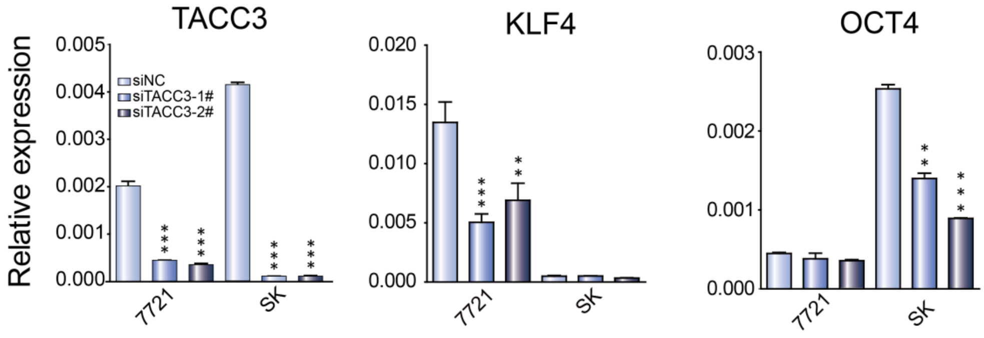

Supplementary Figure4: Knockdown of TACC3 suppressed SCTFs (KLF4&OCT4)

The effect of TACC3 knockdown on theexpressions of stem cell transcription factors, normalized by β –actin. As the figure showed, the suppress trend of KLF4 was obvious in 7721 and OCT4 was obvious in SK. We concluded that silencing TACC3 suppressed SCTF markers, but the effect depended on cell lines.

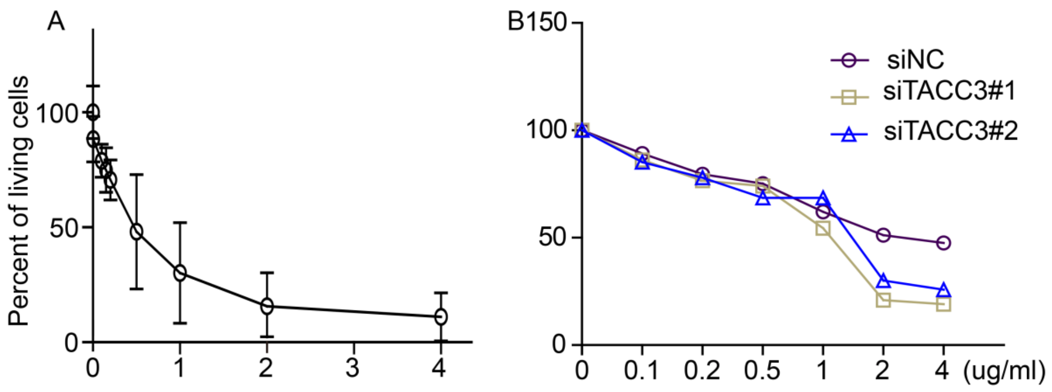

Supplementary Figure5: Knockdown of TACC3 may improve the chemotherapy sensitivity

(A) The IC50 of epirubicin in 7721 (0.5uM) (B) 7721 HCC cells were cultured for 48 hr with various concentrations ofepirubicin. The percentage of viable cells relative to control was determined by MTT assay (mean + SE). TACC3 knockdown increased cellular sensitivity to epirubicin.
